# Eye Development under the control of SRp55/B52-Mediated Alternative Splicing of *eyeless*

**DOI:** 10.1371/journal.pone.0000253

**Published:** 2007-02-28

**Authors:** Weronika Fic, François Juge, Johann Soret, Jamal Tazi

**Affiliations:** Institut de Génétique Moléculaire de Montpellier (IGMM), UMR 5535, Université de Montpellier II, Centre National de Recherche Scientifique (CNRS), Montpellier, France; Centre de Regulació Genòmica, Spain

## Abstract

The genetic programs specifying eye development are highly conserved during evolution and involve the vertebrate *Pax-6* gene and its *Drosophila melanogaster* homolog *eyeless* (*ey*). Here we report that the SR protein B52/SRp55 controls a novel developmentally regulated splicing event of *eyeless* that is crucial for eye growth and specification in *Drosophila*. B52/SRp55 generates two isoforms of *eyeless* differing by an alternative exon encoding a 60-amino-acid insert at the beginning of the paired domain. The long isoform has impaired ability to trigger formation of ectopic eyes and to bind efficiently Eyeless target DNA sequences *in vitro*. When over-produced in the eye imaginal disc, this isoform induces a small eye phenotype, whereas the isoform lacking the alternative exon triggers eye over-growth and strong disorganization. Our results suggest that B52/SRp55 splicing activity is used during normal eye development to control eye organogenesis and size through regulation of *eyeless* alternative splicing.

## Introduction

Alternative splicing enables metazoan genomes to expand their coding capacities through synthesis of different mRNAs from single genes [1]. It has been estimated that approximately 40% and 74% of *Drosophila* and human genes, respectively, encode alternatively spliced pre-mRNAs [2], [3]. It is, therefore, anticipated that alternative splicing participates in the regulation of the gene-expression program that is required for multi-cellular organism development [4], [5]. However, for many genes, the evidence for a change in function due to the generation of alternatively spliced transcripts is based solely on analysis of the mRNA transcripts, with no confirmation that distinct protein isoforms are expressed *in vivo* [6], [7]. Also, isoform-specific targeting has been performed only in few cases to gain insight into how the function and expression of the protein isoforms differ in physiological context [4], [8], [9]. Most of our knowledge about alternative splicing mechanisms has been gleaned from use of model minigene reporters [4], [10], however, much less is known about the capacity of specific splicing factors to influence specific developmental programs.

Among the splicing factors involved in splice site choice, members of the SR (Ser/Arg-rich) family of proteins play a major role [11]–[14]. These proteins constitute a family of splicing factors that are highly conserved in multi-cellular organisms [15], [16]. They have a modular structure that consists of one or two RNA-recognition motifs (RRMs) and a carboxyl (C)-terminal arginine(R)/serine(S)-rich domain (the so-called RS domain). SR proteins participate both in constitutive and alternative splicing by recruiting the general splicing machinery to splicing signals and by binding to regulatory elements in the pre-mRNA [14], [17], [18]. These splicing functions are modulated by antagonistic factors [19]–[21] and phosphorylation of serine residues located within the RS domain [14], [22]. SR proteins can, therefore, affect usage of alternative 5′ or 3′ splice sites in a concentration-dependent manner [23]–[27].

The physiological relevance of members of the SR protein family became apparent with the realization that they are essential for cell viability and/or animal development in different model systems [15], [21], [28]–[32]. The specific role(s) that individual SR proteins play in specific physiological and developmental processes, however, is (are) largely unknown. The *Drosophila* eye organogenesis provides an excellent system to identify general molecular mechanisms regulating specific developmental steps [33]. Interestingly, several well characterized genes that function at the early steps of eye development encode different splice variants. Among these genes, *eyeless* (*ey*), *dachshund* (*dac*), *eyes absent* (*eya*), and *eygone (eyg)* have the capacity to activate the program that is responsible for eye formation when their expression is ectopically targeted to imaginal discs of *Drosophila* other than the eye [33], [34]. Homologs of these genes also play a primordial role in vertebrate eye development, revealing that evolutionarily conserved genes are involved in determining the different eye types in the various metazoan phyla [34]. They are, therefore, considered as master genes of eye development.

Here we report that the SR protein B52/SRp55 controls a critical splicing event of *eyeless* pre-mRNA that changes the biochemical and physiological properties of the encoded protein isoforms. Binding of B52/SRp55 to exonic sequences in the first intron of *eyeless pre-mRNA* allows production of a novel developmentally regulated protein isoform with additional 60 amino-acids immediately upstream from the DNA binding domain (paired domain). Over-production of this novel isoform in the eye results in small eye phenotype, whereas the canonical Eyeless induces eye over-growth. These results show for the first time that a splicing factor, namely, B52/SRp55 directs eye size through production of two alternatively spliced isoform of *Eyeless* a master control gene for morphogenesis.

## Results

### B52 gain of function alters eye development

**Figure 1:**
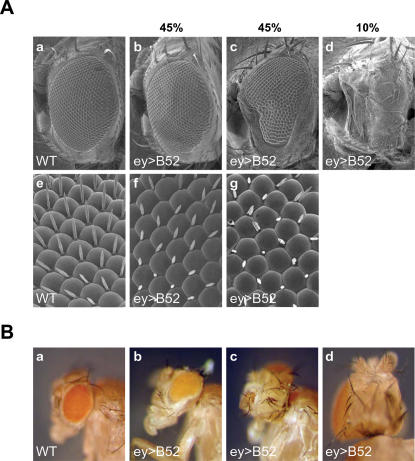
Over-expression of B52 under the control of the *ey-Gal4* transgene impairs eye development. (A) Scanning electron microscope images of wild-type flies (a, e) and UAS-B52/+; ey-gal4/+flies (ey>B52) (b–d, f and g). Photographs were taken at 140× (a–d) and 900× (e–g). (B) ey>B52 flies display variable eye phenotypes ranging from small reductions to absence of the eye (a–d).

B52 activity is critical for *Drosophila* development as both loss of function and gain of function lead to lethality [35]. By using the GAL4/UAS binary expression system [36] to drive expression of B52 in a tissue-specific manner, it was possible to obtain viable adults harboring phenotypes in the eye (GMR-gal4 driver) or in bristles (HS-gal4 and sca-gal4 drivers) [21]. B52 Over-expression under the control of the *eyeless-GAL4 (ey-GAL4)* driver, which directs expression to the primordial eye disc in embryos and the imaginal eye disc [37], profoundly affected eye development (Fig. 1). When *UAS-B52* females are mated to *ey-gal4* males, only 40% of the progeny reached the adult stage. 45% of surviving flies had reduced eye size (Fig. 1A, panel c and Fig. 1B, panel b) and 10% lacked one eye (Fig. 1A, panel d and Fig. 1B, panels c and d). Strikingly, these defects are reminiscent of phenotypes associated with *eyeless* mutations that disrupt an eye-specific regulatory element of the *eyeless (ey)* gene [38], suggesting that B52 Over-expression may alter *ey* expression. The observed phenotypes appear to be specific for B52 Over-expression because relatively weak defects of retinal development or no phenotypes are observed with another SR protein, dASF, when overexpressed by the same driver using *UAS-dSF2* lines (data not shown). Both western-blotting and semi-quantitative reverse transcription (RT)-PCR of RNA from eye imaginal disc confirmed this assumption and demonstrated that in transgenic flies the over-expression of B52/SRp55 did not exceed 2 fold compared to wild type (not shown). Thus, moderate excess of B52/SRp55 in developing eyes gives rise to the same phenotype as low levels of eyeless gene expression. However, quantitative RT-PCR failed to demonstrate dramatic changes in *eyeless* total mRNA levels in B52/SRp55 transgenic flies compared to wild type. The expression levels of other master genes involved in eye formation were also not detectably changed.

### B52 levels affect alternative splicing of developmentally regulated eyeless gene

**Figure 2:**
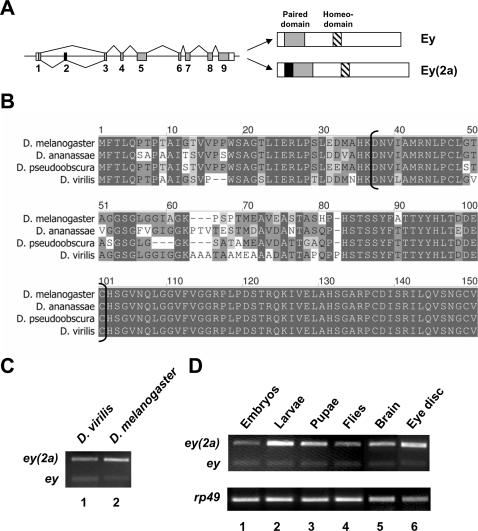
Conservation of eyeless exon 2 alternative splicing. (A) Genomic organization of the *D. melanogaster eyeless* gene. Alternative inclusion of exon 2 (left panel, black box) generates the Ey(2a) isoform that contains 60 additional amino acids (right panel, black box) upstream from the paired domain (right panel, grey box) compared to the canonical Ey isoform. The homeodomain is shown as the hatched box. (B) Sequence comparison of the first 150 amino acids of the Ey(2a) isoform in four *Drosophila* species. *D. melanogaster* exon 2 sequence was used to blast the first intron of the ey gene in the three other species. Predicted protein sequences were deduced from the following genomic scaffolds: AE003843 (*D. melanogaster*), AAPP01017013 (*D. ananassae*), CH475402 (*D. pseudoobscura*), CH940665 (*D. virilis*). The protein sequences derived from exon 2 are between the brackets. (C) *eyeless* exon 2 is alternatively spliced in *D. virilis* and *D. melanogaster*. RT-PCR with primers in exon 1 and 3 of *ey* was performed on total RNA from *D. virilis* (lane 1) and *D. melanogaster* (lane 2) larvae. (D) *ey* alternative splicing during *D. melanogaster* development. RT-PCR with primers in exon 1 and 3 of *ey* was perfomed on total RNA from *D. melanogaster* at various developmental stages and in different tissues of third instar larvae.

Failure to find quantitative changes in *eyeless* expression levels prompted us to test whether qualitative changes occurred that could account for the observed phenotypes associated with B52 over-production. While most studies about *eyeless* gene function have considered only one isoform encoded by this gene, one report in the literature alluded to isolation of a cDNA that encodes a longer isoform [37]. The latter isoform is also present among ESTs described in the fly Database and corresponds to inclusion of an exonic sequence contained in the first intron of the previously described *eyeless* gene (Fig. 2A). Sequence comparison between four *Drosophila* species revealed that the intron/exon structure of the eyeless gene is highly conserved and all contain similar exonic sequences in the first intron (not shown). This exon codes for protein fragment that is higly conserved at the beginning and final third with a central region that is less conserved (Fig. 2B). RT-PCR analysis of RNA from *D. melanogaster* and *D. virilis* revealed that this exon is alternatively spliced in these two fly species (Fig. 2C). Together these observations suggest that the longer splice variant carries out an essential function that depends on alternative splicing. Here we refer to the isoform including exon 2 as *ey(2a)*.

**Figure 3:**
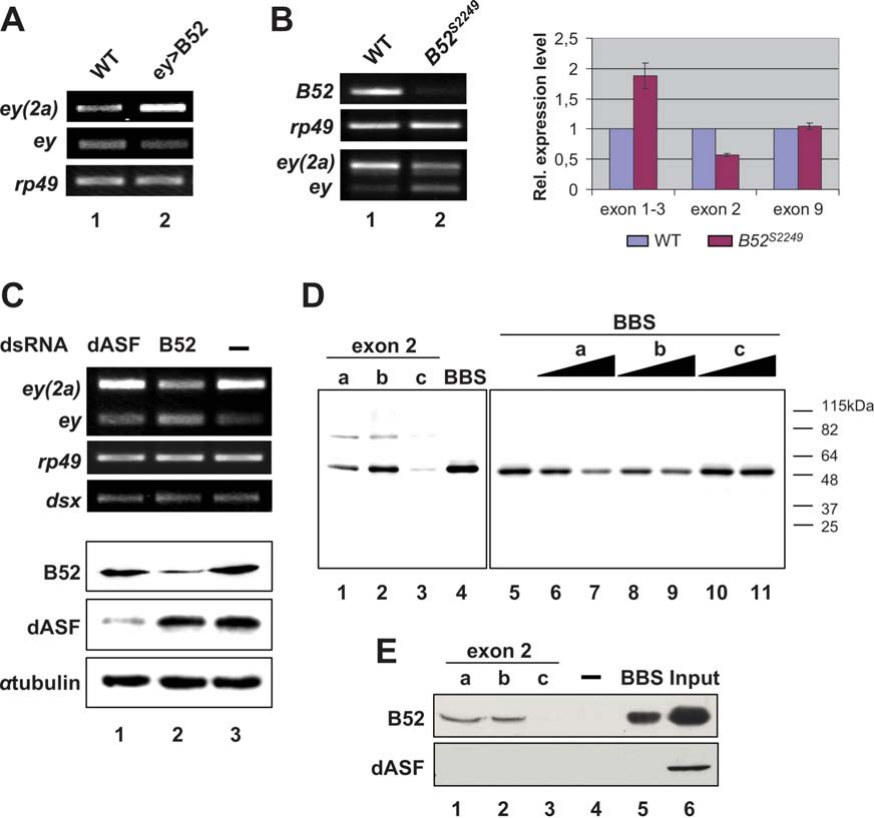
B52 regulates alternative splicing of *eyeless in vivo*. (A) RT-PCR analysis of *eyeless* expression in wild type (lane 1) and ey>B52 (lane 2) eye imaginal discs at the third instar larval stage. RT-PCR was performed with primers specific for each isoform. (B) RT-PCR analysis of *ey* exon 2 inclusion in wild-type (lane 1) and *B52^s2249^* mutant (lane 2) second instar larvae. The right panel corresponds to quantitation of *ey* exon 2 splicing in *B52^s2249^* normalized to wild-type (WT). RT-PCR was performed in triplicates with primers specific for *ey* isoform (exon 1–3), *ey(2a)* isoform (exon 2), or both (exon 9); and expression was normalized to the RP49 level. The expression level in WT was arbitrary set up to 1. Quantitation with primers specific for *ey* exon 9 showed that the global level of eyeless expression does not change in *B52^s2249^* mutant background. (C) SL2 cells were treated with dsRNA against dASF (lane 1) or B52 (lane 2) or untreated (lane 3), and analyzed by RT-PCR (top panel) or western blotting (bottom panel). (D) Cross-linking of exon 2 sequences to B52. Radiolabelled probes corresponding to contiguous sequences of exon 2, named a, b and c were incubated in Kc nuclear extracts and exposed to UV light. A high affinity binding site for B52 (BBS) was used as a positive control. Autoradiography of the SDS-PAGE (left panel) shows that probes a (lane 1) and b (lane 2), as well as BBS (lane 4), predominantly cross-link a 52 kDa protein. Cross-linking of BBS to 52 kDa protein was efficiently competed by increasing amounts of unlabelled probes a (right panel; lanes 6 and 7) and b (lanes 8 and 9) but not c (lanes 10 and 11). (E) RNA probes a (lane 1), b (lane 2), c (lane 3) and BBS (lane 5) were covalently bound to beads and incubated with Kc nuclear extract. After washing, beads were loaded on an SDS-PAGE and transferred to nitrocellulose. The membrane was probed with anti-dASF (panel dASF) and anti-B52 sera (panel B52). Beads alone (lane 4) and Kc nuclear extract alone(lane 6) were run as controls.

Previous results identified the spliced form *ey(2a)* in embryos and larvae [37], however, the levels of individual e*y* and e*y(2a)* isoforms were not compared at the different developmental stages. Therefore, mRNA products of *eyeless* gene were examined by RT-PCR and normalized to ribosomal *Rp49* mRNA at different developmental stages (Fig. 2D). Both larvae and pupae contained larger amounts of *ey(2a)* than *ey* mRNA (Fig. 2D, lanes 2–3). In contrast, embryos and adult flies had almost equal levels of both types of mRNA (Fig. 2D, lanes 1 and 4). To determine whether these two isoforms are expressed in different tissues or are co-expressed in the same tissues, we analysed their distribution in two larval tissues. In third instar larvae, *ey* is expressed in the eye disc and in the brain. RT-PCR analysis on these two isolated tissues revealed that both *ey* isoforms are co-expressed in these tissues (Fig. 2D, lanes 5 and 6). Albeit, both tissues contained larger amounts of *ey(2a)* than *ey* mRNA. Moreover, cultured SL2 cells expressed both isoforms (see below, Fig 3C), suggesting that they are expressed in the same cells.

Given the existence of *ey* exon 2 alternative splicing in the eye imaginal disc, we asked whether B52 over-expression under the control of *ey-gal4*, which triggers the phenotypes depicted in Fig. 1, can perturb exon 2 inclusion. RT-PCR analyses, using primers that discriminate exon 2 inclusion (Fig. 3A, panel ey(2a)) from exon 2 skipping (Fig. 3A, panel ey), on WT and ey>B52 eye imaginal discs showed that exon 2 inclusion is increased by B52 over-expression (Fig. 3A, all panels compare lanes 1 and 2), suggesting that B52 is involved in exon 2 splicing. To further investigate this hypothesis, we asked whether B52 loss of function can affect *ey* exon 2 alternative splicing. To this end, we took advantage of a *B52^s2249^* mutant that contains a *P{lacW}* transgene inserted 16 nt downstream from the *B52* transcription start site (*Flybase*). *B52^s2249^* homozygous larvae die at the first- and second-instar larval stage ([39] and present study), similar to the *B52^28^* null larvae [30]. Absence of B52 mRNA was confirmed by RT-PCR analysis of RNA extracted from living *B52^s2249^/B52^s2249^* larvae (Fig. 3B, lane 2). RT-PCR analyses with *eyeless*-specific primers revealed, however, that B52 depletion is correlated with a reduction in the *ey(2a)* mRNA isoform and with a parallel increase in the expression level of *ey* mRNA (Fig. 3B, lower left panel compare lanes 1 and 2). Quantitation with primers that distinguish between the two isoforms demonstrated a two fold increase in the *ey* isoform concomitant with a two fold decrease the e*y(2a)* isoform (Fig. 3B, right panel). These results demonstrate that B52 contributes to the production of *ey(2a)* isoform in whole second-instar larvae.

### Exon2 of eyeless contains B52 binding sequences

To further confirm the involvement of *B52* in regulating *eyeless* alternative splicing, we employed RNA interference in *Drosophila* SL2 cells. We observed that SL2 cells express both *ey* isoforms in a ratio similar to that observed in brain and eye tissues (Fig. 3C). Cells were incubated with *B52* double-stranded RNA, and the level of B52 protein was determined by western blot analysis (Fig. 3C, panel B52). As a control, cells were treated in parallel with *dASF*-specific dsRNA (Fig. 3C, panel dASF). Following six days of treatment, each dsRNA efficiently and specifically depleted the corresponding protein (Fig. 3C, compare panels B52 and dASF to panel α-tubulin). RT-PCR analyses of dsRNA-treated cells showed that B52 depletion triggered a significant decrease in *ey(2a)* mRNA levels with a concomitant increase in *ey* mRNA levels (Fig. 3C, upper panel lane 2), whereas dASF depletion had no effect (Fig. 3C, upper panel lane 1). Depletion of either B52 or dASF, however, did not affect the expression of a specific isoform of *doublesex* (Fig. 3C, panel *dsx* lanes 1–3), known to be regulated by SRp20, another member of the SR protein family. Altogether our results show that varying the B52 level *in vivo* modulates exon 2 inclusion and provides strong evidence that B52, but not another SR protein, is required for *ey* exon2 inclusion.

Sites on RNA that bind B52 with high affinity and specificity (BBS) have previously been described [40]. When RNA aptamers containing multi-mers of these BBS were expressed in transgenic flies under the same genetic driver of B52 over-production, they fully restored wild type phenotypes, implying that B52 binds to its target sequences *in vivo* to regulate alternative splicing. To test whether exon 2 of *eyeless* contains sequences recognized by B52, UV crosslinking experiments were performed under splicing conditions using Kc cells nuclear extracts as a source of B52 protein and three probes corresponding to contiguous sequences of exon 2, named (a), (b) and (c), which cover its entire length. Radiolabeled probes (a) and (b) bound to a ∼52 kDa band (Fig. 3D, lane 1–2), which was also detected with a radiolabeled probe corresponding to the B52 high affinity binding site established by SELEX [40] that was used as positive control (Fig. 3D, lane 4), making it very likely that the protein corresponded to B52. The identity of the protein was further confirmed using RNA affinity selection procedure on (a), (b) and (c) fragments, where B52 was detected by anti-B52 antibodies (Fig. 3E). Both (a) and (b) but not (c) RNA fragments are able to bind B52 (Fig. 3E, panel B52 lanes 1–3). Neither fragment, however, showed binding to dASF which, like B52, contains two RRMs (Fig. 3E, panel dASF, lanes 1–3). Consistent with these results, competition experiments showed that the (a)- and the (b)-exon 2 sequences competed with the crosslinking of B52 high affinity binding site (BBS) whereas (c)-type sequence did not (Fig. 3D, lanes 5–11), confirming that both (a) and (b) sequences behave as B52 binding sites. These data provide further evidence that B52 regulates *eyeless* exon 2 inclusion through specific binding of exon 2 sequences.

### High level of Ey(2a) and Ey differentially affect eye growth

**Figure 4:**
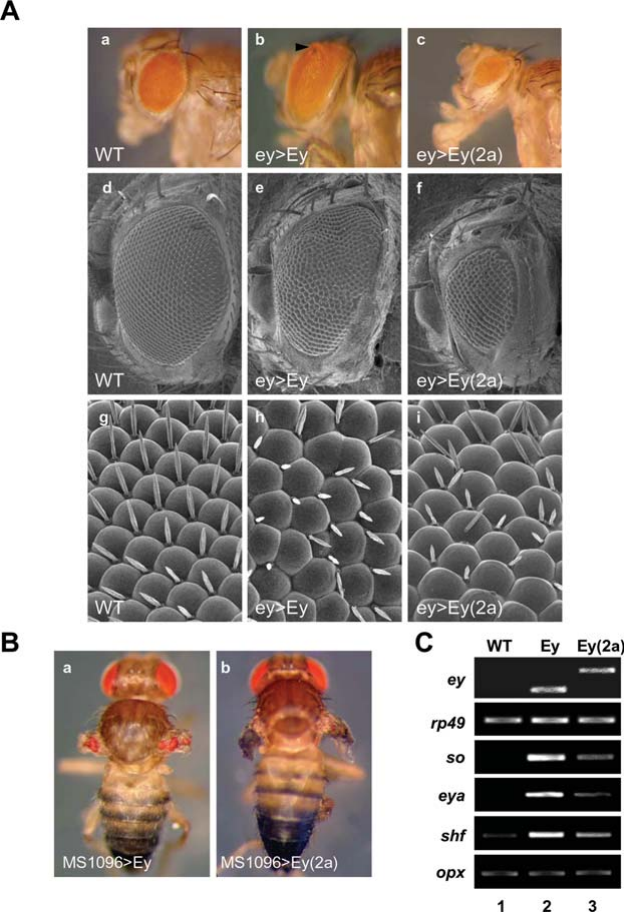
Over-expression of Ey(2a) and Ey isoforms leads to different phenotypes. (A) Representative eye phenotypes obtained after expression of Ey (panel b) or Ey(2a) (panel c) isoforms under the control of ey-Gal4, compared to wild-type flies (panel a). Ey isoform expression induces strong disorganization of the ommatidia lattice (compare panels e and d). Ommatidia appear of variable size with possible fusion between them, as observed in (panel h). ey>Ey flies often display local overgrowth in the eyes (arrowhead in panel b). Expression of the Ey(2a) isoform only reduces the size of the eye (panels c and f) with moderate disorganization of the omatidia lattice (panel i). (B) Expression of Ey (panel a) and Ey(2a) (panel b) isoforms under the control of *MS1096*. (C) Ectopic expression of Ey and Ey(2a) in the wing induces expression of downstream target genes at different levels. RT-PCR analyses were performed to measure the expression of *eyeless* (panel ey), the ribobosomal *Rp49* (panel rp49), *Sine oculis* (panel so), *eyes absent* (panel eya), *shifted* (panel shf) and *Optix* (panel opx) mRNAs in wing discs from wild type (lane 1) MS1096>Ey (lane 2) and MS1096>Ey(2a) (lane 3) third instar larvae.

Because high levels of B52 in the eye are expected to result in increased concentration of Ey(2a) compared to Ey isoform, we were interested to determine the functional consequences of over-producing Ey(2a) on eye development and/or morphogenesis. To this end, we expressed either *ey* or *ey(2a)* cDNAs with the UAS/Gal4 system. Reproducibly, when expressed in the eye disc under the control of the *ey-gal4* driver, Ey(2a) induced formation of small eyes that are the result of reduced number of ommatidia (Fig. 4A, panels c and f). However, all the structures of the ommatidia were present (panel i), implying that Ey(2a) does not interfere with normal process of differentiation of the eye but limits its size. In contrast, expression of the Ey isoform led to more variable phenotypes ranging from a small reduction and disorganization of the eye (Fig. 4A, panels b, e and h, and [41]) to over growth (Fig. 4b). Quantization of these results indicated that small changes in the amount (1.5 to 2 fold) of either isoform were responsible for the observed phenotypes. Given that B52 also regulates splicing in a dose-dependent manner, these results confirmed our finding that B52 finely tunes eye organogenesis by controlling the amount of these splice variants.

The *ey* gene is a master control gene for eye morphogenesis, because the Ey isoform of the gene has the ability to induce ectopic eye structures in several imaginal discs [42], [43]. Therefore, we decided to study the potential of Ey(2a) to induce ectopic eyes by using the Gal4 system to target its expression to imaginal discs where it is normally not transcribed. Comparison of phenotypes induced by MS1096, which allows specific expression of either Ey or Ey(2a) in the wing disc [44], revealed that Ey(2a) was less efficient to trigger ectopic eye structures in the adult wings than Ey (Fig. 4B, compare panels a and b). The eye structures reproducibly were smaller in size with Ey(2a) in comparison to those obtained with the Ey isoform. This observation was further confirmed using the *dpp*-Gal4 driver, which is expressed in all imaginal discs. Again, more pronounced eye morphogenesis was obtained with the Ey isoform than with Ey(2a) where only small foci of unstructured ommatidia were observed (data not shown).

### Ey(2a) is less potent than Ey in activating transcription of target genes and in binding to cognate DNA sequences

The above results are consistent with the hypothesis that Ey and Ey(2a) have distinct function(s) during eye morphogenesis. As several genes are under the control of the *ey* gene to switch on the eye development pathway, it is conceivable that Ey and Ey(2a) isoforms differ in their ability to activate target genes. To test this hypothesis, we determined the expression level of specific target genes after ectopic expression of either isoform in the wing disc. We considered well-characterized direct targets of Ey, *eyes absent (Eya)* and *shifted (shf)*, which were examined using *in situ* hybridization, gel shift assays, and reporter analysis [45], [46]. We also examined expression of *Sine oculis (so)*, a subordinate regulatory gene that mediates *ey* gene activation [45]–[47]. As a negative control, we used *optix* (*opx*) whose transcription is not induced by Ey [48]. RT-PCR analysis demonstrated that while both *ey(2a)* and *ey* isoforms were overexpressed to similar extent (Fig. 4C, panel ey), induction of ey-target genes mediated by Ey(2a) was lower compared with Ey (Fig. 4C, compare lanes 2 and 3 of each panel). As expected neither isoforms stimulated transcription of *optix* gene (Fig. 4C, panel opx), confirming the specificity of induction of the direct target genes.

**Figure 5:**
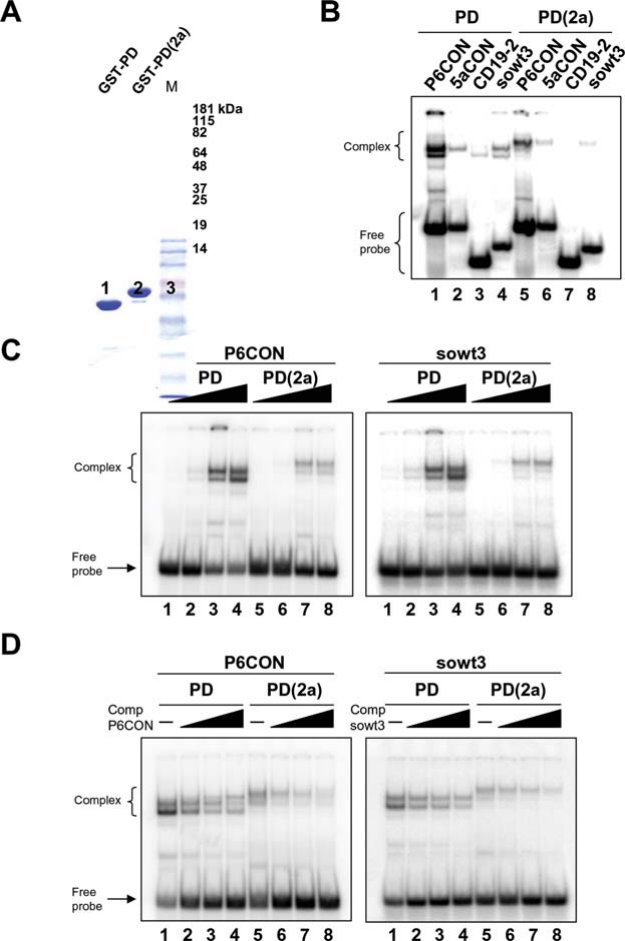
*In vitro* DNA binding activities of Ey and Ey(2a) paired domains. (A) SDS-PAGE of GST-PD and GST-PD(2a) fusion proteins that were expressed and purified from bacteria. (B) EMSA performed with GST-PD (lanes 1–4) or GST-PD(2a) (lanes 5–8) in the presence of four established consensus sequences P6CON (lanes 1 and 5), 5aCON (lanes 2 and 6), CD19-2 (lanes 3 and 7) and sowt3 (lanes 4 and 8) for Ey or its human homolog PAX6 or PAX6(5a) isoform. See text for details. (C) EMSA of P6CON (left panel) and sowt3 (right panel) probes in the presence of increasing amounts of GST-PD (1 ng, lane 1; 5 ng, lane 2; 50 ng, lane 3; 200 ng, lane 4) and GST-PD(2a) (1 ng, lane 5; 5 ng, lanes 6, 50 ng, lane 7; 200 ng, lane 8) proteins. (D) EMSA of P6CON (left panel) and sowt3 (right panel) probes with 50 ng of GST-PD (lanes 1–4) or GST-PD(2a) (lanes 5–8) in the presence of (1 fold, lanes 2 and 6; 2 fold, lanes 3 and 7; 10 fold, lanes 4 and 8) of unlabelled corresponding probes or without competitor (lanes 1 and 5).

The differences in expression of the target genes could be explained if Ey(2a) and Ey isoforms exhibited different affinities for target DNA sequences. The Ey protein contains two DNA binding domains, a paired domain (PD) and a paired-type homeodomain (HD) both of which are capable of binding specific DNA sequences. Only PD, but not HD, protein has been shown to be essential for eye development [49]. Given that Ey(2a) has additional amino-acids immediately upstream from its paired domain, it is possible that this modification affects its binding ability and/or specificity. To directly test these possibilities, the two Ey paired domains (denoted PD and PD(2a)) were expressed in *E. coli* as gluthatione S-transferase (GST) fusion proteins (Fig. 5A), and their DNA binding abilities were examined by electrophoretic mobility shift assay (EMSA). In the presence of a large excess (200 fold) of cold competitor, the extended paired domain PD(2a) did not bind to CD19-2, the high affinity binding site for the shorter eyeless paired domain [50], (Fig. 5B, compare lanes 3 and 7). PD(2a) also bound less efficiently to other paired domain recognition sites, including sowt3, a sequence found upstream of *so* and demonstrated to regulate its transcription by Eyeless [51] (Fig. 5B, compare lanes 4 and 8, and Fig. 5C, right panel for quantitative comparison). Weak binding affinity was also demonstrated for P6CON, a high affinity binding site for Pax6 [52], the human homolog of Eyeless (Fig. 5B, compare lanes 1 and 5, and Fig. 5C, left panel for quantitative comparison). The specificity of binding of recombinant proteins to these sequences was observed, however, when increasing amounts of unlabeled probes were added and showed to compete with themselves in the presence of large excess of competitor (Fig. 5D, left and right panels).

Since P6CON sequence was also refractory for binding the paired domain of Pax6(5a), a Pax6 isoform with a 14-amino-acid insertion in the paired domain that arises by alternative splicing (Epstein et al., 1994), it was important to determine whether PD(2a) has the same specificity as Pax6(5a). EMSA assays were, therefore, performed with 5aCON, a sequence that selectively binds Pax6(5a) paired domain [52]. As previously observed, 5aCON did not have a high affinity for PD(2a) compared to PD (Fig. 5B, compare lanes 2 and 6). Furthermore, while 5aCON changed the EMSA binding profile of Pax6(5a) paired domain [52], no changes in gel shift were observed with PD(2a). Together, these results suggest that the 60 additional amino-acids at the N-terminal part of the paired domain of *eyeless* weaken its DNA binding activity.

## Discussion

In this work, we show that B52, an SR protein splicing factor, controls the production of two alternatively spliced isoforms *ey* and *ey (2a)* of *eyeless*, a master control gene for eye morphogenesis. Our data not only establish the mechanistic link between a splicing factor and a critical component of eye development, but also demonstrate the significance of this regulation *in vivo*. To date, only the *ey* isoform has been extensively studied, and its ectopic expression shown to induce functional eyes on the legs, wings, and antennae of the fly [42]. Here we demonstrate that *ey* and *ey(2a)* isoforms generated by alternative splicing are co expressed throughout *Drosophila* development. The importance of this splicing event for eye formation is underscored by its conservation among several *Drosophila* species and its involvement in changing the DNA binding properties of a hallmark feature of Pax gene family, the PD domain. Unlike Ey, Ey(2a) has an impaired ability to trigger efficient *ey* target gene expression and to bind efficiently to DNA cognate sequences. Intriguingly, our data further suggest that *ey(2a)* is apparently acting in a dominant-negative fashion toward *ey*. Over-production of *ey(2a)* in the eye causes a small eye phenotype, whereas, under the same conditions *ey* overproduction produces local eye over-growth. Thus, during eye formation, small changes in the *ey/ey(2a)* equilibrium may ensure a correct number of ommatidia and thereby control eye morphogenesis. In this context, it may be relevant that the activity of the *eyeless* gene is adjusted by a splicing event producing two isoforms with antagonistic activities rather than a transcription factor, like *twin of eyeless* (*toy*), that may turn it on and off in a more restricted way [53].

Our data also provide support for the idea of a direct connection between pre-mRNA splicing and eye development being conserved from *Drosophila* to mammals [33], [38]. As in vertebrates, where the single *Pax6* gene produces two alternatively splicing isoforms, referred to as *Pax6* and *Pax6(5a)* that exhibit distinct functions [52], the *eyeless* gene also encodes two isoforms with different transcriptional properties. In the case of the *Pax 6* gene, alternative splicing generates two transcripts that differ in the inclusion of 14 amino acids encoded by the additional exon 5a [52]. This insertion occurs immediately N-terminal of an α-helix that is important for the recognition of specific DNA sequences by the PD domain and thereby profoundly alters its DNA-binding activity [52]. When tested on the well-known bipartite paired domain recognition sequences both *ey(2a)* and *Pax6(5a)* PD domains fail to bind [53]; this study). However, Pax6(5a) but not Ey(2a) could interact with the highly specialized recognition sequence 5aCON ([52], this study), suggesting that Ey(2a) might have a different specificity from Pax6(5a). Alternatively, additional amino acids N-terminal to PD domain of Ey(2a) may mask the PD domain and thus prevent its interaction with target DNA sequences. Future experiments are needed to establish the structure of the Ey(2a) PD domain with its additional N-terminal amino acids and to more definitively test whether Ey(2a) activates target genes other than those recognized by Ey and/or mediates assembly of different transcriptional complexes to exhibit distinct transcriptional activation properties.

Our experiments suggest that one of the functions mediated by B52 during *Drosophila* eye development is to attenuate the effect of an over-expression of *ey* that could be detrimental for eye morphogenesis. This proposal stems from the ectopic expression experiments showing that over-expression of B52 is associated with partial or complete loss of the eye, a phenotype that is equivalent to inactivation of an eye-specific enhancer of the *eyeless* locus by transposon insertion [37]. Further support comes from B52 depletion experiments showing a two fold reduction of *ey(2a)* and concomitant increase of *ey* mRNA levels. However, the e*yeless* alternative splicing event was not identified in large scale analysis of alternatively spliced pre-mRNAs that are aberrantly regulated in B52-deficient tissue culture cells [54]. Given that previous studies have used a robust system to select alternative splicing events and only weak expression of eyeless was detected in SL2 cells (present study), it is likely that the two fold changes in the *ey/ey(2a)* ratio was below the threshold to be detected by Blanchette et al. [54]. Thus, genetic and biochemical analyses like the ones described in this paper appear to be essential to decipher the function of specific isoforms, whose levels are moderately altered during *Drosophila* development.

The exact mechanism by which B52 influences alternative splicing of *eyeless* pre-mRNA is still unknown. *In vitro* data showed that B52 binds directly the alternative exon 2 and mediates its inclusion. However, computational scanning of the target exonic sequence for previously reported SELEX consensus RNA binding sequence recognized by B52 [55], [40], failed to reveal any match to this sequence, suggesting that B52 interacts with a set of distinct RNA sequences to regulate the *eyeless* splicing event. It is also possible that, as with other RNA binding proteins [14], [17], *eyeless*-regulated splice sites require the formation of large, multi-protein complexes compatible with the requirement for a higher order of complexity, rather than a single RNA-protein interaction. Identification of partners assembled in these complexes will be informative about mechanism(s) leading to tissue-specific regulation of *eyeless* alternative exon 2. We cannot completely rule out the possibility, however, that deregulation of *eyeless* alternative splicing in both *B52^-^* larvae and in *B52* RNAi-mediated knock down in SL2 cells may be due to an indirect effect. But failure to observe similar splicing phenotype associated with either over expression or depletion of another SR protein, dASF, further supports the idea that eyeless alternative splicing is specifically mediated by direct interaction of B52 with exon 2 sequences.

B52 deficiency does not seem to induce major defects in growth and differentiation of the eye disc during larval stage [39] and does not abolish eyeless pre-mRNA splicing, but rather makes a specific contribution to its regulation during eye morphogenesis. During the larval period, a wave of differentiation and patterning called the morphogenetic furrow (MF) progresses from posterior to anterior across the disc epithelium [56]. Anterior to the furrow are the dividing, undifferentiated progenitor cells; immediately behind the furrow, cells form differentiating clusters; and more posterior, these clusters acquire their final differentiated state [34]. *Eyeless* is expressed throughout the undifferentiated progenitor cells at the anterior part of the eye imaginal disc, and its expression is down-regulated in the MF where cells are held in G1 [34]. B52 may act directly on the progression of the MF, as it has been recently shown to maintain the G_1_/S block *in vivo* by specific regulation of the repressor function of dE2F2 [39]. It is possible that B52-mediated *eyeless* splicing is regulated at the entry of the MF to control the number of ommatidia founder cells. Further insights into specific regulation of *ey* pre-mRNA splicing by B52 will likely require identification of signaling pathways that modulate the level of B52 and/or activity at the MF. Among these signalling pathways Hedgehog (Hh) [57], Dpp, a secreted molecule [58] and the Notch pathway are known to be important for eye development [59].

## Materials and Methods

### Drosophila stocks

*eyeless* cDNA E10 cloned in pUAST vector was provided by W. Gehring (Biozentrum der Universitat Basel, Basel, Switzerland). To obtain the pUAST-Ey(2a) construct, *ey* cDNA E10 was cloned into the XhoI/XbaI sites of the pSP72 vector (Promega). A 493 nt NcoI/NaeI fragment was replaced by a 672 bp NcoI-NaeI fragment containing exon 2a. This 672 bp NcoI-NaeI eyeless cDNA fragment was amplified with high fidelity Pfu polymerase from larval cDNAs and subcloned into the TOPO–TA PCRII vector (Invitrogen). PCRII-Ey(2a) was entirely sequenced to verify its integrity. The 2.8 kb XhoI-XbaI fragment containing *ey(2a)* cDNA was inserted into the pUAST vector. pUAST-Ey(2a) and pUAST-Ey plasmids were used to transform w^1118^ flies according to standard protocols [58]. The transposon integration sites were mapped to individual chromosomes by standard crosses using balancer stocks. Five independent UAS-Ey and UAS-Ey(2a) transgenic lines were analysed in all experiments. Gal4 lines and the *B52^s2249^* line were obtained from the Bloomington *Drosophila* Stock Center. All crosses were reared at 25°C on standard medium.

### RNA extraction and RT-PCR

Total RNA was extracted from cultured cells or larval tissues using TRI Reagent (Sigma Aldrich) and treated with RNase-free DNase I. cDNA was synthesized with First Strand cDNA kit (Amersham Pharmacia) using an oligo-(dT) primer. PCR products were separated by agarose gel electrophoresis and visualized by ethidium bromide staining. Sequences of the primers are available upon request.

### RNAi

RNA interference treatments were performed in SL2 cells according to Worby et al. (2001). Cells were treated with double-stranded RNA corresponding to the entire coding sequence of B52/Srp55 or dASF at day 1 and harvested on day 6 for western and RT-PCR analysis.

### Antibodies

The Anti-dASF and Anti-B52 sera against the peptide GSYRGGNRNDRSRD corresponding to aa 85 to 98 of *Drosophila* dASF, and to the peptide KNGNASPDRNNESMDD at the C-terminal end of B52, respectively were raised in rabbits by Eurogentec.

### GST constructs and gel shift

The N-terminal region of Eyeless corresponding to the paired domain (PD) or to the PD with the region encoded by exon 2a were cloned into the NotI –SalI sites of pGEX-5x vector to give the GST-PD or GST-PD(2a) constructs respectively. GST-fusion proteins were produced in *E. coli* strain BL21(DE3) and were purified according to standard protocols. Double-strand DNA probes were obtained by mixing complementary oligonucleotides and were radiolabelled at their 5′-end with γ-^32^P ATP. Binding assays contained approximately 0.5 ng of DNA probe and varying concentrations (0.5–200 ng) of purified GST-PD or GST-PD(2a) proteins. Gel shift reactions were performed in 25 mM Hepes (pH 7.6), 10% glycerol, 100 mM KCl, 1 mM DTT 1% NP40, 0,1% BSA, 200× DNA competitor. DNA-protein complexes were resolved on 6% polyacrylamide gels in 0.5× TBE buffer. Complexes were revealed by autoradiography. Sequences of the probes are available upon request

### Cross-linking and affinity purification

Cross-linking experiments were performed with probes corresponding to three fragments (a, b and c) covering the entire 180 bp exon 2a. Each 60 bp fragment, obtained by PCR, was cloned into the BamHI/EcoRI sites of pGEM2 vector (Promega). The control probe BBS was obtained by PCR performed on genomic DNA from UAS-BBS-5.12 flies (generous gift from John Lis). This fragment containing 2 high affinity binding sites for B52 was cloned into pGEM2. Radiolabelled RNA probes a, b, c and BBS were transcribed *in vitro* with SP6 or T7 RNA polymerase, 1 µg of the suitable linearized plasmids, 5 µM [α-^32^P]UTP and 5 µM [α-^32^P]GTP (800 Ci/mmol) in 25 µl reaction mixtures according to the manufacturer's instructions (Promega). For UV cross-linking experiments Kc nuclear extract was pre-incubated for 15 min at 30°C in buffer containing 10 mM Tris (pH 7.5), 10% glycerol, 0.1 mM EDTA, 0.75 mM ATP, 25 mM creatinine phosphate, 1 mM MgCl_2_, 250 ng tRNA, 1 mM DTT, 40 U RNasin, 30 ng/µl BSA, then radiolabelled RNA was added and incubated 15 min. Reactions were irradiated for 20 min on ice with UV light (254 nm) at a distance of 3 cm. The RNA was digested with RNase A and T1 for 30 min at 37°C. Cross-linked proteins were separated on 10% SDS-PAGE. Dried gels were exposed to Phosphorimager.

Binding experiments with RNA probes immobilized on agarose beads were performed essentially as described by Caputi et al [60]. Substrate RNAs for bead immobilization were synthesized *in vitro* by using the SP6 or T7 Ribomax large scale RNA production system (Promega). Following incubation with the splicing mix; bound proteins were eluted by addition of sample buffer, heated for 5 min at 90°C, and separated on 12% SDS-PAGE. Western-blot was performed using antibodies against B52 and dASF.
